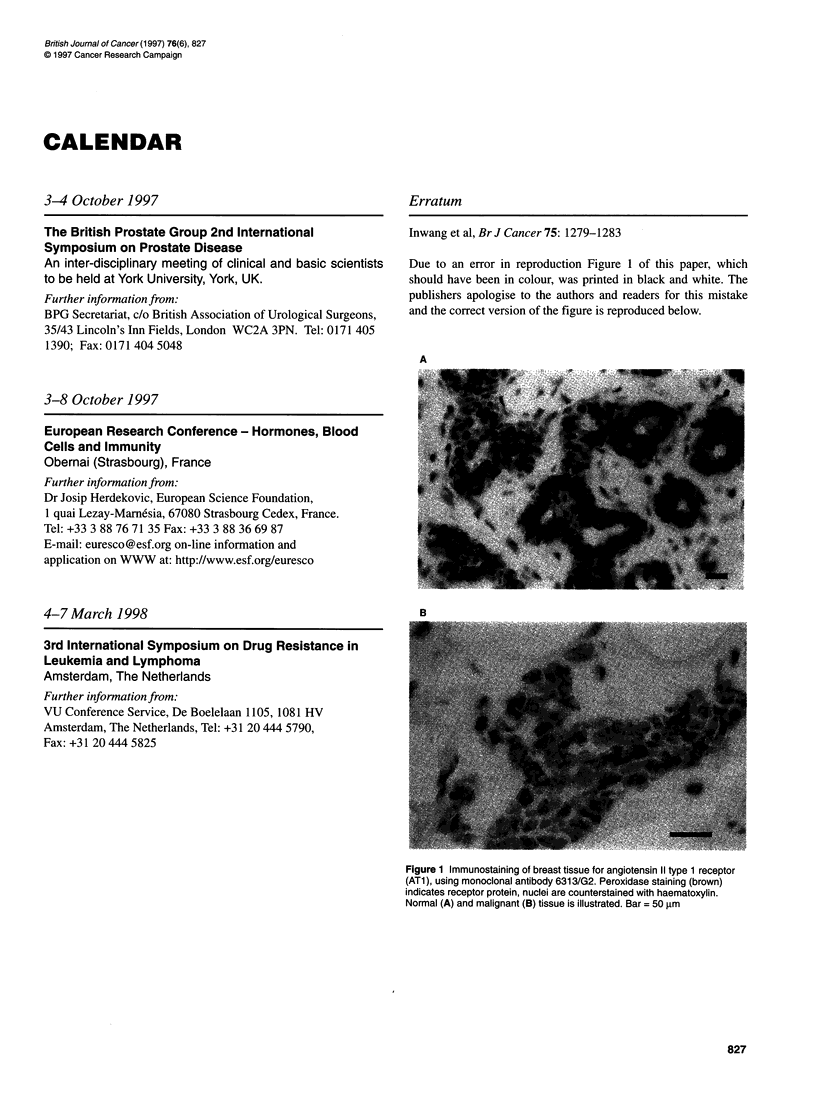# Calendar

**Published:** 1997

**Authors:** 

## Abstract

**Images:**


British Journal of Cancer (1997) 76(6), 827
© 1997 Cancer Research Campaign

### CALENDAR

### 3–4 October 1997

**The British Prostate Group 2nd International Symposium on Prostate Disease**
An inter-disciplinary meeting of clinical and basic scientists to be held at York University, York, UK.

*Further information from:*
BPG Secretariat, c/o British Association of Urological Surgeons, 35/43 Lincoln's Inn Fields, London WC2A 3PN. Tel: 0171 405 1390; Fax: 0171 404 5048

### 3–8 October 1997

**European Research Conference – Hormones, Blood Cells and Immunity**
Obernai (Strasbourg), France

*Further information from:*
Dr Josip Herdekovic, European Science Foundation,
1 quai Lezay-Marnésia, 67080 Strasbourg Cedex, France.
Tel: +33 3 88 76 71 35 Fax: +33 3 88 36 69 87
E-mail: euresco@esf.org on-line information and application on WWW at: http://www.esf.org/euresco

### 4–7 March 1998

**3rd International Symposium on Drug Resistance in Leukemia and Lymphoma**
Amsterdam, The Netherlands

*Further information from:*
VU Conference Service, De Boelelaan 1105, 1081 HV Amsterdam, The Netherlands, Tel: +31 20 444 5790,
Fax: +31 20 444 5825